# Recurrent Staphylococcus capitis Prosthetic Valve Endocarditis Presenting With ST-Segment Elevation Myocardial Infarction: A Case Report

**DOI:** 10.7759/cureus.22732

**Published:** 2022-03-01

**Authors:** Islam M Shatla, Fady Banno, Ain Ejaz, Angel Lopez Candales

**Affiliations:** 1 Internal Medicine, University of Missouri Kansas City School of Medicine, Kansas City, USA; 2 Internal Medicine, Ascension Genesys Hospital, Grand Blanc, USA; 3 Internal Medicine, University of Missouri Kansas City, Missouri, USA; 4 Cardiovascular Medicine Division, University of Puerto Rico School of Medicine, San Juan, USA

**Keywords:** staphylococcus capitis, endocarditits, st-segment elevation myocardial infarction, prosthetic valve endocarditis, recurrent staphylococcus capitis

## Abstract

We report a case of ST-elevation myocardial infarction (STEMI) due to septic emboli secondary to *Staphylococcus capitis *endocarditis in a 32-year-old male patient with a past medical history of infectious endocarditis requiring mechanical aortic, mitral and tricuspid valve replacement presented with sharp chest pain and shortness of breath. Electrocardiogram demonstrated an acute inferior STEMI.

Coronary angiography revealed occlusion of the terminal left anterior descending (LAD) artery associated with a large apical wrap-around segment exhibiting TIMI 0 flow. Primary angioplasty was not performed given the distal location of the embolus. Clinical suspicion for septic or thrombotic coronary artery embolism was high given the patient’s history of mechanical valve prosthesis and in the setting of sub-therapeutic INR. Transesophageal echocardiography revealed a new mobile echodensity on the mitral prosthesis consistent with vegetation. *S. capitis* was isolated from blood cultures, confirming the diagnosis of endocarditis.

*S. capitis* is a rare cause of prosthetic valve endocarditis and should remain in the differential of septic coronary artery embolism among patients with features of infectious endocarditis.

## Introduction

Prosthetic valve endocarditis (PVE) is a rare but rather serious and potentially life-threatening complication after valve replacement [1]. PVE significantly differs from native valve endocarditis in terms of both morbidity and mortality. Two main issues are critical when facing potential cases of PVE, establishing a diagnosis, and implementing effective treatment. Even though there is no characteristic clinical presentation that would be unique for PVE, patients might simply present with fever and loss of appetite, symptoms that otherwise are seen in the post-operative period and the patients might be neglected and not recognized early on. Surely, they can present with new or worsening heart failure, a new or changed heart murmur, heart block, or any other new conduction abnormality (LBBB), while others might have a cerebral embolic event or myocardial infarction [2]. It has been reported that PVE accounts for 20% of all cases of endocarditis with an incidence ranging from 0.3% to 1.2% per patient-year while the most common causative organisms are *Staphylococcus aureus* in early PVE (less than 12 months after valve replacement) and coagulase-negative Staphylococci in late PVE (greater than 12 months after valve replacement) [3].

*Staphylococcus capitis* is a coagulase-negative Staphylococci (CoNS) which is part of the normal flora of the human face and scalp [4]. CoNS are well known to possess the ability to form biofilms leading to their virulence in the development of device-associated infections [5]. But *S. capitis* in particular has a lower ability to attach foreign body surfaces as compared to other CoNS, which makes it a rare microorganism causing PVE [6]. In addition, it has not been reported to be associated with coronary artery embolism.

We report a case of ST-elevation myocardial infarction (STEMI) due to septic emboli secondary to *S. capitis* endocarditis. Due to the rarity of cases previously reported with *S. capitis*, we feel that this case will be a useful addition to the existing literature. It is important to identify the offending organism in any given case so that effective treatments can be effectively implemented in a timely fashion and for expansion of guideline-based management with emphasis on prophylaxis or prevention strategies if warranted in the future.

## Case presentation

**Figure 1:**
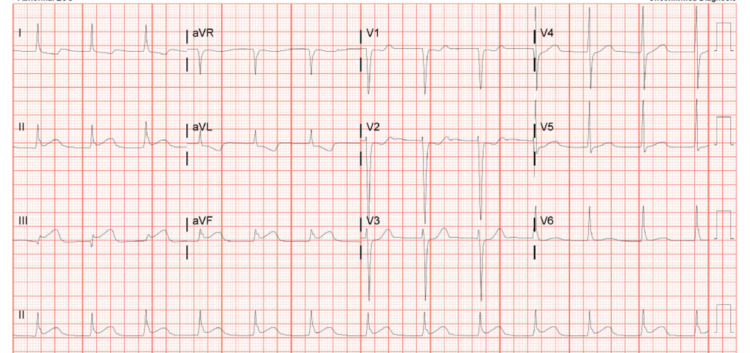
EKG showing ST-elevation MI in inferior leads EKG: Electrocardiogram; MI: Myocardial Infarction

A 32-year-old male patient with a past medical history of infectious endocarditis requiring mechanical aortic, mitral, and tricuspid valve replacement in 2011 presented with sharp chest pain and shortness of breath. The patient was afebrile, had a blood pressure of 100/44 mmHg, pulse 76 beats/min, and chest auscultation revealed a systolic murmur and metallic click over the upper left sternal border. Electrocardiogram demonstrated an acute inferior STEMI (Figure 1).

**Figure 2:**
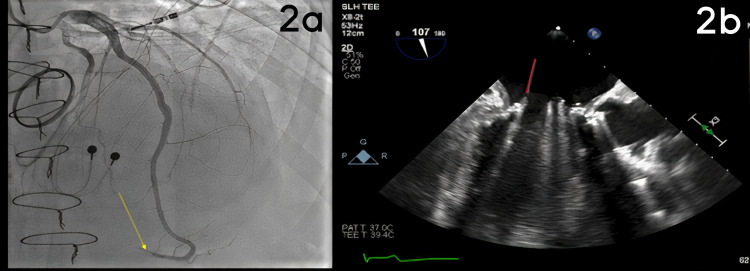
(a) Yellow arrow points to distal LAD thrombus in LAO cranial view. (b) Red arrow points to vegetation on the mitral valve in TEE. LAD: Left Anterior Descending Artery; LAO: Left Anterior Oblique; TEE: Transesophageal Echocardiogram

Coronary angiography revealed occlusion of the terminal left anterior descending (LAD) artery associated with a large apical wrap-around segment exhibiting TIMI 0 flow (Figure 2a). Primary angioplasty was not performed given the distal location of the embolus. Clinical suspicion for septic or thrombotic coronary artery embolism was high given the patient’s history of mechanical valve prosthesis and in the setting of sub-therapeutic INR. Transesophageal echocardiography (TEE) revealed a new mobile echo density on the mitral prosthesis consistent with vegetation (Figure 2b). *S. capitis* was isolated from blood cultures, confirming the diagnosis of endocarditis. The patient was treated with IV vancomycin for six weeks according to organism susceptibility testing, was anticoagulated, and discharged without incident. A repeat blood culture came back negative after three days of vancomycin. The patient was stable on discharge. Follow-up TEE showed resolution of echo dense mass on the mitral prosthesis.

## Discussion

Coagulase-negative staphylococci, mainly *Staphylococcus epidermidis* is well documented in the literature to cause PVE. Its role in native valve endocarditis is much smaller (<10%) [7,8].

*S. capitis* is a coagulase-negative, novobiocin-sensitive, aerobic, and hemolysis-positive. It lacks the alkaline phosphatase activity that is seen in *S. epidermidis* [9]. Coagulase-negative staphylococci (CoNS) are constituents of our normal skin flora and have been shown to colonize foreign materials in the human body. *S. capitis* mainly colonize the skin of the scalp, ears, neck, and face and it accounts for approximately 5% of coagulase-negative isolates that cause pneumonia, urinary tract infection, cellulitis, and bacteremia [9-15]. The adhesion ability of *S. capitis* to foreign surfaces is lower compared to most CoNS; however, *S. capitis* as a cause of infective endocarditis is well documented in the literature but not that common.

Additionally, there is a small number of cases that involve prosthetic valves [16-18]. In these cases, early removal of the prosthetic valve appears to be effective. Other cases with *S. capitis* native valve endocarditis are successfully treated with antibiotics alone.

It is well documented in the literature that *S. aureus* is the most common cause of PVE, followed by the CoNS *Enterococcus* and *Streptococcus viridans* [19]. The ability of CoNS to adhere and grow on prosthetic devices is very important in causing disease. However, *S. capitis* has a weaker adhesion capability to smooth surfaces compared to other CoNS [20,21]. The adhesion ability of *S. capitis* to prosthetic valves is due to biofilm production as well as secretion of exoenzymes [13,21]. Of note, *S. capitis* has not been reported in the literature to be associated with coronary artery embolism.

Our patient is a young male who had infectious endocarditis requiring mechanical aortic, mitral, and tricuspid valve replacement, presented with sharp chest pain and shortness of breath. *S. capitis* was isolated from blood cultures, confirming the diagnosis of endocarditis. The patient was treated with IV vancomycin for six weeks according to organism susceptibility testing, was anticoagulated, and discharged without incident. A repeat blood culture came back negative after three days of vancomycin.

Treating PVE remains a challenge. PVE has a mortality rate of 21%-28.4% [20]. However, most of the patients with *S. capitis* PVE reported in the literature as well as our current case showed improvement after antibiotic treatment only. Only a few cases required surgical intervention with antibiotics (Table 1).

**Table 1:** S. capitis PVE reported in the literature with treatment included. PVE: Prosthetic Valve Endocarditis

Case no	Age, gender	Valve affected	Management	Reference
1	72, M	Mitral	Vancomycin + Gentamycin	[11]
2	53, M	Mitral	2 weeks of amoxicillin + netilmicin followed by 4 weeks of ceftriaxone	[17]
3	63, M	Tricuspid	Cloxacillin	[14]
4	29, M	Mitral	Penicillin + Gentamicin	[15]
5	62, M	Mitral	Surgery and Vancomycin + Gentamicin. Later treated with Penicillin and Gentamicin and then Rifampin and Pefloxacin.	[15]
6	70, M	Aortic	Nafcillin + Gentamicin	[10]
7	73, M	Mitral	Ampicillin + Gentamicin + Cloxacillin	[7]
8	46, M	Aortic	Vancomycin + Rifampin	[22]
9	35, M	Aortic	Vancomycin + Rifampin	[22]
10	35, M	Aortic	Vancomycin + Rifampin	[23]
11	79, F	Aortic	Surgery + IV Vancomycin and oral minomycin	[24]
12	79, F	Aortic	Surgery + Vancomycin and Rifampin	[24]
13	76, M	Aortic	Surgery + IV teicoplanin and linezolid	[24]
14	68, F	Mitral	Surgery + IV Vancomycin and Gentamycin	[24]

## Conclusions

*S. capitis* is a rare cause of prosthetic valve endocarditis and should remain in the differential of septic coronary artery embolism among patients with features of infectious endocarditis. Correct identification of coagulase-negative Staphylococci is important to help in the decision to manage the disease either with medical or combined medical and surgical intervention.
